# Radial artery perforation: when a friend turns against you

**DOI:** 10.1186/s43044-019-0015-1

**Published:** 2019-09-11

**Authors:** Mostafa Elwany, Roberto Adriano Latini, Gaetano Di Palma, Bernardo Cortese

**Affiliations:** 1 Interventional Cardiology, ASST Fatebenefratelli-Sacco, Fatebenefratelli Hospital, Corso di Porta Nuova, 23, 20121 Milan, Italy; 2 Faculty of Medicine, University of Alexandria, Alexandria, Egypt; 3 Interventional Cardiology, San Carlo Clinic, Milano, Italy; 4 Fondazione G. Monasterio CNR-Regione Toscana, Pisa, Massa, Italy

**Keywords:** Radial artery perforation

## Abstract

**Background:**

Radial artery approach is currently the most common access site for coronary angiography and percutaneous coronary intervention. It rarely results in complications, improves patient comfort, and reduces the duration of hospitalization.

**Case presentation:**

A 91-year-old woman presented to our institution with ST-segment elevation myocardial infarction (STEMI). The right radial access was chosen for the performance of percutaneous coronary intervention. After the introduction of 6 F sheath, there was difficulty in the advancement of 0.035 J wire that was exchanged with a Terumo hydrophilic wire. After the procedure and before sheath removal, radial arteriography was done and revealed perforation. Protamine sulfate was administered and prolonged balloon inflation was attempted but failed to seal the perforation, so a 7-F-long vascular sheath was inserted to internally tamponade the vessel, and the patient was sent to the coronary care unit for monitoring. Over the next 3 days, serial radial angiographies were done revealing the persistence of the perforation, and on the fourth day, angiography revealed multiple thrombi. Thrombus aspiration was done using Pronto V4 extraction catheter (Vascular Solutions, USA) and was followed by the deployment of a covered stent. The stent was dislodged and successfully snared. Finally, the perforation was sealed spontaneously and there were no signs of intra-arterial thrombi.

**Conclusion:**

Despite the very low complication rate of radial approach, the interventional cardiologist should be aware of any possible complication, and how to avoid or, eventually, manage it.

## Background

Radial artery approach is currently the most common access site for coronary angiography and percutaneous coronary intervention (PCI) since it results in fewer local vascular complications than transfemoral approach. This approach rarely results in complications, improves patient comfort, and reduces the duration of hospitalization [1].

## Case presentation

**Fig. 1:**
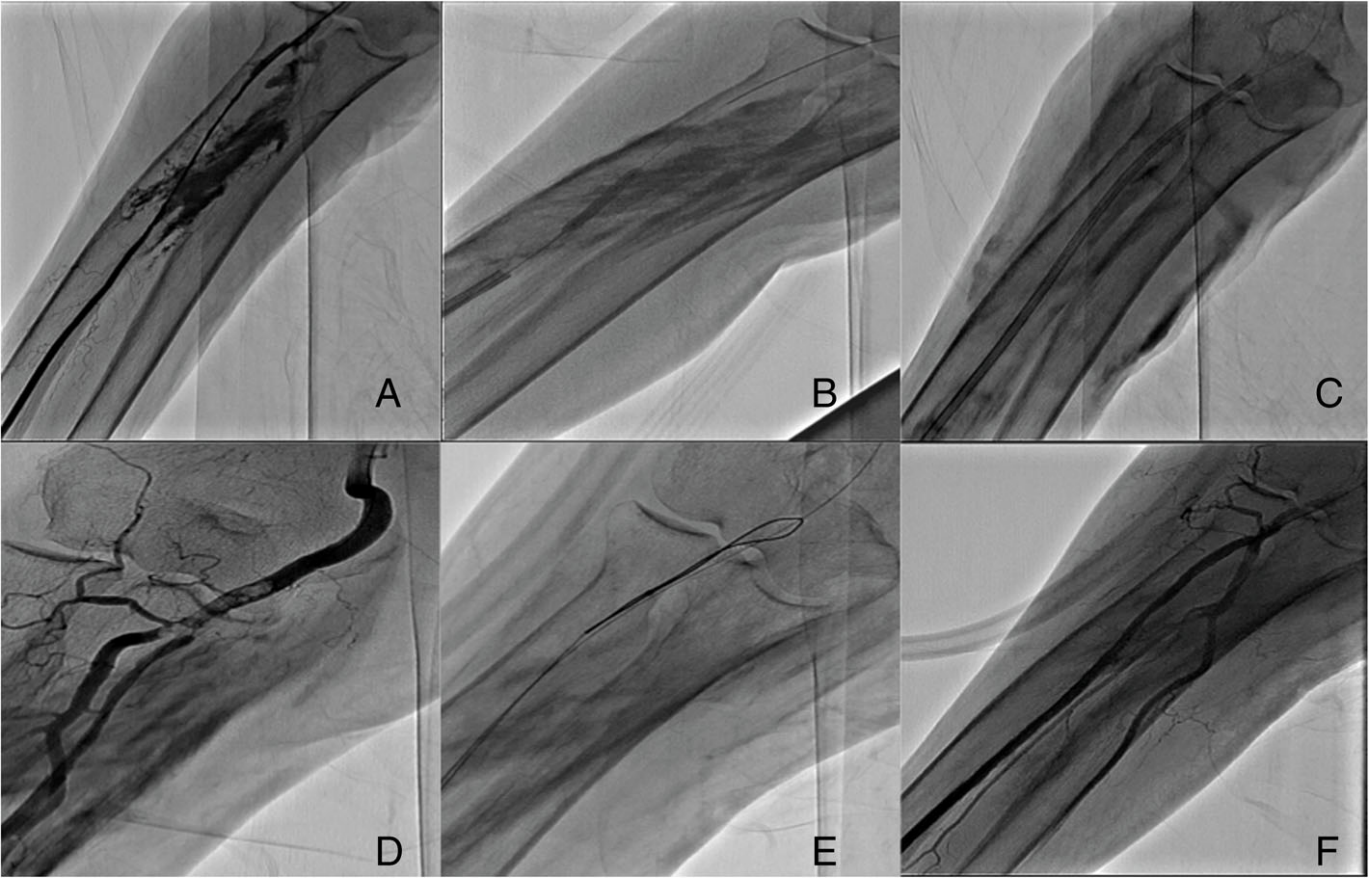
**a** Angiogram of the radial artery showing radial artery perforation. **b** Prolonged balloon inflation. **c** A 7-F-long vascular sheath was inserted to internally tamponade the vessel. **d** After 4 days, angiography revealed multiple thrombi. **e** Successful snaring of the covered stent. **f** Final result after the perforation was sealed

A 91-year-old woman presented to our institution with ST-segment elevation myocardial infarction (STEMI). The right radial access was chosen for the performance of percutaneous coronary intervention (PCI). After the introduction of 6 F sheath, there was difficulty in the advancement of 0.035 J wire that was exchanged with a Terumo hydrophilic wire (0.035 × 180) which was advanced easily to the aortic root. Coronary angiography was done and revealed tortuous coronary arteries without significant lesions. Before sheath removal, radial arteriography was done and revealed perforation (Fig. 1a). Protamine sulfate (1 mg per 100 USP units of heparin) was administered intravenously to reverse the dose of heparin (70 U/kg), and APTT was monitored 15 min after the dose. This was followed by prolonged balloon inflation 2.5/3.0 (Fig. 1b). The perforation was not sealed so a 7-F-long vascular sheath was inserted to internally tamponade the vessel, and the patient was sent to the coronary care unit for monitoring of the forearm hematoma and the distal pulses (Fig. 1c). Over the next 3 days, serial radial angiographies were done which revealed the persistence of the perforation. After 4 days, angiography revealed multiple thrombi (Fig. 1d), and thrombus aspiration was done using Pronto V4 extraction catheter (Vascular Solutions, USA) and was followed by the implantation of a covered stent, which was dislodged, mostly secondary to under expansion, following another run of thrombus aspiration. The stent was successfully snared (Fig. 1e). Finally, the perforation was sealed spontaneously and there were no signs of intra-arterial thrombi (Fig. 1f).

## Discussion

Radial artery perforation is a rare complication (< 1%) and often leads to forearm hematoma [2]. Conservative management including neutralization of heparin, crossing with a wire, and deployment of either a long sheath or guide catheter across and external compression by sphygmomanometer cuff may help in sealing the perforation [3]. In some rare cases, in case of persistance of the perforation, balloon angioplasty or use of covered stent has been described [4]. This case is unusual in that the perforation did not seal conservatively at first together with the heavy thrombus burden that was managed by thrombus aspiration, which is rarely used in the management of such cases.

## Conclusion

Despite the very low complication rate of radial approach, the interventional cardiologist should be aware of any possible complication, and how to avoid or, eventually, manage it [5].

## Data Availability

Not applicable

## Abbreviations

PCI: Percutaneous coronary intervention
STEMI: ST-segment elevation myocardial infarction
APTT: Activated partial thromboplastin time
